# Integrated nano-optomechanical displacement sensor with ultrawide optical bandwidth

**DOI:** 10.1038/s41467-020-16269-7

**Published:** 2020-05-15

**Authors:** Tianran Liu, Francesco Pagliano, René van Veldhoven, Vadim Pogoretskiy, Yuqing Jiao, Andrea Fiore

**Affiliations:** 1 Institute for Photonic Integration, Eindhoven University of Technology, P.O. Box 513, 5600MB Eindhoven, The Netherlands; 2 nanoPHAB, Groene Loper 19, Postbus 513, 5612 AP Eindhoven, The Netherlands

**Keywords:** Integrated optics, Optical sensors

## Abstract

Optical read-out of motion is widely used in sensing applications. Recent developments in micro- and nano-optomechanical systems have given rise to on-chip mechanical sensing platforms, potentially leading to compact and integrated optical motion sensors. However, these systems typically exploit narrow spectral resonances and therefore require tuneable lasers with narrow linewidth and low spectral noise, which makes the integration of the read-out extremely challenging. Here, we report a step towards the practical application of nanomechanical sensors, by presenting a sensor with ultrawide (∼80 nm) optical bandwidth. It is based on a nanomechanical, three-dimensional directional coupler with integrated dual-channel waveguide photodiodes, and displays small displacement imprecision of only 45 fm/Hz^1/2^ as well as large dynamic range (>30 nm). The broad optical bandwidth releases the need for a tuneable laser and the on-chip photocurrent read-out replaces the external detector, opening the way to fully-integrated nanomechanical sensors.

## Abstract (web summary)

On-chip mechanical sensing platforms require tuneable lasers with narrow linewidth and low spectral noise, making read-out challenging. Here, the authors report a nanomechanical sensor with 80nm bandwidth, displacement imprecision of 45 fm/Hz^1/2^ as well as a dynamic range greater than 30 nm with integrated photodetectors.

## Introduction

Micro- and nano-optomechanical systems (NOMS) have been widely investigated for the sensing of displacement, force and mass, and find applications in atomic-force microscopy (AFM), photoacoustic spectroscopy, accelerometry, and mass sensing^1,2^. Their optical read-out provides the benefits of high resolution, high operating frequency and relative noise immunity. As a prototypical example, the conventional AFM read-out method, based on position-sensitive detection of cantilever deflection, combines high resolution and dynamic range, but is relatively bulky and cannot be easily integrated on a chip. The demand for size and cost scaling is pushing the development of integrated read-out solutions. Recently, nanophotonic cavities have shown the potential for high-resolution displacement sensing on an integrated chip^3–12^. sensitivity is realized by micro-rings^3,8^, micro-disks^4–7^ and photonic crystal cavities^9–12^ with optical quality factors up to hundreds of thousands. However, the resonant nature of the cavity response intrinsically limits the dynamic range and the optical bandwidth, putting stringent requirements on the read-out system. In order to appreciate the corresponding limitations, we note that a commercial AFM system combines a displacement imprecision in the tens of fm/Hz^1/2^ range, together with a maximum displacement amplitude of a few hundred nanometers using a simple red read-out laser^13,14^. In contrast, resonant-cavity-based nano-optomechanical systems can have ultrahigh sensitivity below fm/Hz^1/2^ but at cost of a limited dynamic range of a few nanometers, and require a high-performance tuneable laser with picometer linewidth and low-frequency noise.

On the way to practical integrated nanomechanical sensing solutions, two key problems must be overcome: On one hand, the measurable displacement range must be increased (to tens or hundreds of nanometers) for robust surface scanning measurement; on the other hand, the interrogation and read-out system must be massively simplified, with full integration of laser, sensor and detector being the ultimate goal. While the dynamic range can be partially improved by reducing the optomechanical coupling constant in a resonant-cavity sensor^4^, the second requirement points to a different solution, namely the use of non-resonant sensing, e.g., based on position-sensitive detection or on interferometers with large free spectral range. While resonant systems offer the highest response to displacement, this can be partially be compensated by higher laser power as they are more immune to heating and dynamical backaction^15^. Additionally, laser frequency noise becomes less critical, which, together with the large optical bandwidth, reduces the requirements of complexity on the read-out laser. So far only a few nano-optomechanical systems without cavities have been reported, e.g., based on side- or top-coupled Mach–Zehnder interferometers^16,17^, and suspended waveguide with a free end^18^. However, their displacement imprecision has remained limited to ≫ 1 pm/Hz^1/2^, with no demonstration of Brownian motion sensing. A broadband sensor with 40 fm/Hz^1/2^ imprecision based on nanocantilevers was demonstrated on silicon-on-insulator in 2009^19^, but it required high on-chip power ∼7 mW due to the relatively low displacement sensitivity, and did not feature integrated photodetectors. We further note that previous sensing structures were demonstrated in silicon, which makes it difficult to integrate a read-out laser.

Here we present an integrated sensor that satisfies all the requirements for chip-based displacement measurement. It employs a nanomechanical sensor, a 3D directional coupler, which allows transducing vertical displacement into a change of the relative transmission of two output waveguides. It features a low displacement imprecision of 45 fm/Hz^1/2^ with a large displacement dynamic range (>30 nm) and ultrabroad optical linewidth (>80 nm). This sensitivity is achieved with an optical power-on-chip (~0.3 mW), over an order of magnitude smaller than that in^19^. Additionally, two Indium Phosphide (InP) photodiodes for the readout and an electrostatic actuator are integrated with the sensor, enabling static control and dynamic electrical excitation of the mechanical resonator. The use of an InP platform further opens the way to the integration of lasers for the read-out, potentially resulting in fully-integrated optical sensors.

## Results

### Design of the displacement sensor and integrated photodetector

**Fig. 1:**
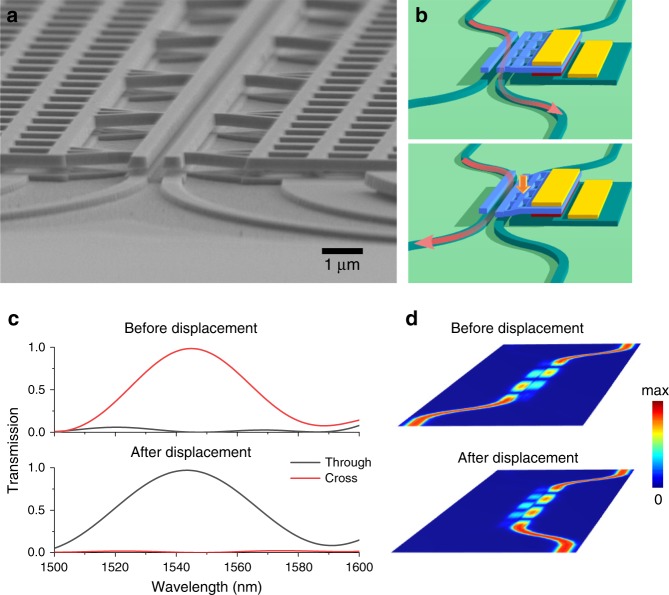
Displacement transducing by nanomechanical directional coupler. **a** Scanning electron microscope (SEM) image of the nanomechanical directional coupler used as a transducer. **b** Schematic illustration of light passing through the direction coupler before (up) and after (down) actuation. **c** Simulated transmission and **d** simulated electric field distribution (|E|) before and after displacement (55 nm) of an ideal device described in ref. ^20^.

The heart of the transducer is a nanomechanical directional coupler consisting of four evanescently-coupled waveguides, of which the two top ones are suspended (Fig. 1a). In this structure the vertical displacement of a waveguide results in a change in the relative transmission from the two output waveguides. This is obtained by a combination of vertical and horizontal evanescent coupling^20^. The transducing concept is illustrated in Fig. 1b, in the two cases where the system is symmetric (same gap thicknesses – “before displacement”) and after reducing one of the gaps (“after displacement”). The structure is designed in such a way that, before displacement, light coming from one input waveguide excites a superposition of symmetric and anti-symmetric supermodes which, after traveling for a beating length in the directional coupler, interferes constructively at the “cross” output port. A displacement of one suspended waveguide changes the propagation constants of the supermodes and makes the interference destructive, resulting in increased transmission from the other waveguide (“through” port). Figure 1c, d shows the simulated transmission and the electric field distribution of the two cases in a finite-difference time-domain (FDTD) model. A complete change in transmission is predicted with one of the gaps varying from 220 nm to 165 nm. The maximum displacement sensitivity is $\frac{\partial T}{\partial z}$ = 21 µm^−1^, which is ~10 times higher than the previously reported broadband sensor^19^. The bandwidth is comparable to that in ref. ^19^, limited only by the condition of maintaining constructive/destructive interference. For the structures used in this work, the free spectral range is more than 90 nm. Besides broad bandwidth, another advantage of this read-out is that transmission of the two outputs always changes oppositely with displacement. This configuration enables a differential read-out to cancel laser intensity fluctuations.

**Fig. 2:**
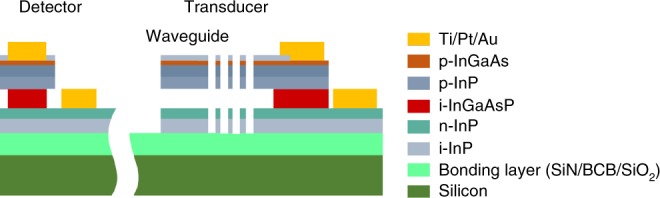
Schematic cross-section of the layer stack of the device. The detector (left) and the transducer/actuator part (right).

The sensor was fabricated on the InP membrane-on-silicon (IMOS) platform. Compared to silicon photonics, this platform allows integration of passive components, lasers and detectors in a micron-thick and high-confinement InP membrane^21^. The waveguides are based on two InP membranes separated by 220-nm-thick InGaAsP (i-Q1.58) sacrificial layer, which is etched in the sensing region to suspend the top membrane, while it is kept and used as absorbing layer in the photodetector section. The top, 330-nm-thick InP membrane is p-doped, with a 20-nm-thick p-InGaAs contact layer, while the bottom membrane consists of a top 50-nm-thick n-doped InP layer and a 220-nm-thick undoped layer. The corresponding p-i-n junction is used to electrostatically actuate the inter-membrane distance in the sensing section, and forms the photodiode in the detection section as shown in Fig. 2. Fabrication consists of two-electron beam lithographies and consecutive dry etching steps to define the waveguides and cantilever, and three lithographies to define the contacts for actuator and readout. The detailed process is described in the method section.

**Fig. 3:**
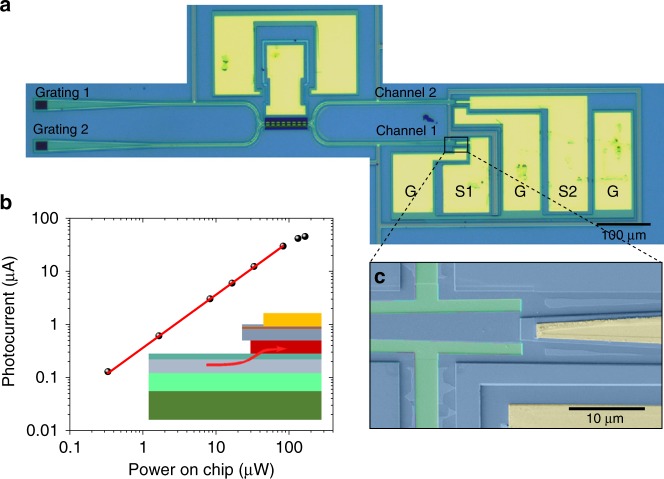
Integrated sensor and response of the photodiode. **a** Optical microscope image of the integrated displacement sensor. G stands for ground, S1 and S2 stand for signal of channel 1 and channel 2. **b** Photocurrent measured on a waveguide photodiode connected directly with a grating coupler at 1540 nm as a function of power on chip. Linear fit of the photocurrent with power under 100 µW is indicated by red line. Inset: Schematic of the integrated photodiode. Red arrow marks the direction of incoming light. **c** SEM image of the integrated photodiode in false colors.

Figure 3a shows the optical microscope image of the whole sensor composed of transducer, actuator, photodiodes and grating couplers. One of the suspended waveguides can be electrostatically actuated in the out-of-plane direction by the reverse-biased p-i-n junction, for tuning or dynamically driving the device. The two output waveguides of the directional coupler guide the light into the two on-chip waveguide photodiodes (Fig. 3c). In the photodiode section the waveguides are tapered to a larger width so that sufficient amount of the absorbing quaternary layer is left after the undercut.

We first show the characteristics of the integrated photodiodes. The photocurrent, measured with a 10 kΩ load resistor and zero bias, is plotted in Fig. 3b as a function of the waveguide-coupled power, showing a linear increase below 0.1 mW, and then gradually saturates as the output voltage approaches the built-in voltage of the diode. A responsivity of 0.36 A/W is measured below 0.1 mW under zero bias. A statistical variation of up to 30% in the responsivity is observed on the measured detectors due to nonunifomities in the diodes or the grating couplers. Applying a negative bias could improve the responsivity, however, we chose to work under zero bias to avoid additional noise from the dark current. Part of the light is absorbed by the top metal pad, which also contributes to the loss of responsivity. The saturation photocurrent can be improved by using a lower load resistance.

### Static response of the displacement sensor

**Fig. 4:**
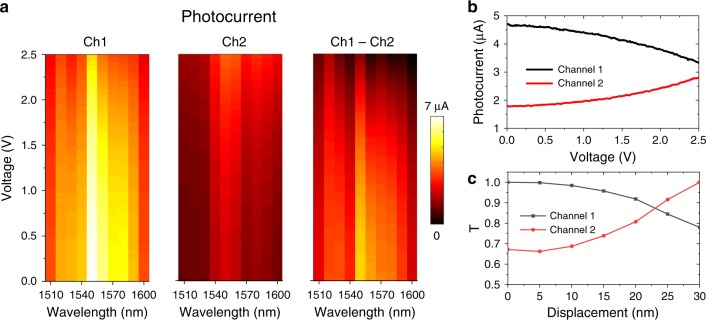
Response of the senor under a static actuation. **a** Photocurrent of the two detectors and their difference, as a function of reverse bias voltage on the actuator, for an on-chip laser power of 0.3 mW. **b** Measured photocurrent at 1540 nm as a function of bias voltage. **c** Simulated transmittance at 1540 nm as a function of the displacement of the suspended waveguide.

By applying a reverse bias from 0 V to 2.5 V on the actuator, we measure the static sensor response of the two channels at different wavelengths from 1510 nm to 1600 nm, as shown in Fig. 4a. The input optical power is 10 mW, and the coupling efficiency from input to the waveguide is about 15 dB, which gives a coupled on-chip power of 0.3 mW. The lower waveguide has a loss of 2.6 dB/mm from roughness and absorption of the doped layer, and the scattering loss from the MEMS cantilever is about 40 dB/mm. In total they contribute to an insertion loss of about 5 dB at the center wavelength, excluding that of the photodiode and the grating coupler. The measured photocurrent in both channels reaches a maximum around wavelength of 1550 nm due to the design of the grating couplers. In the entire measured wavelength range, the modulation of photocurrent due to the actuation is between 25% and 60% of its maximum. The two channels are always modulated in opposite directions as predicted by the model, but the contrast ratio of the photocurrents between the two channels is limited due a mismatch between the length of the coupling region and the critical coupling length. This is due to the processing imperfections in e.g., the etching depth and the slot width between the waveguides. A simulation model using FDTD is built with the parameters from the SEM to calculate the transmittance in the two channels as a function of displacement of one suspended waveguide, which is shown in Fig. 4c. By comparing Fig. 4b, c, we note that the ratio of the photocurrents between the two channels does not match that of the ratio of the simulated transmittance between them, however, the relative changes of each photocurrent are similar to that of the simulated transmittance. We attribute this discrepancy to the difference between the actual critical coupling length and that used in the model, as well as the nonuniformity of the photodiodes.

### Imprecision of the displacement sensor

**Fig. 5:**
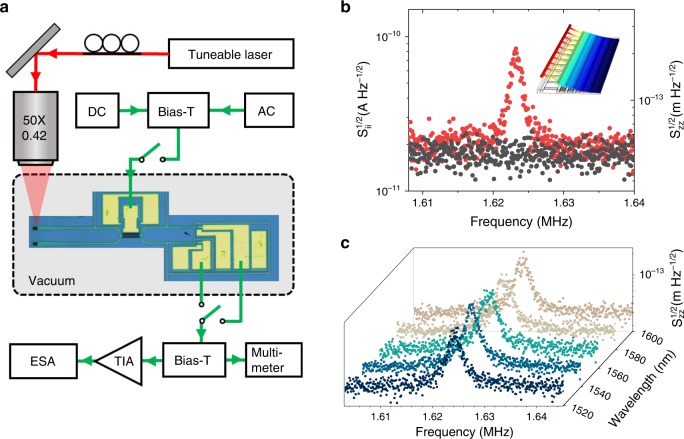
Experimental set-up and the measurement of the thermal noise. **a** Schematics of the thermal noise measurement setup. **b** Photocurrent (left axis) and displacement (right axis) spectral density, measured by the ESA with (red circles) and without (black circles) laser (λ = 1540 nm, on-chip power 0.3 mW, actuator bias voltage 1.5 V), showing a peak corresponding to the Brownian motion of the fundamental mechanical mode. Inset: Calculated mode shape of fundamental mechanical mode. **c** Brownian motion measured at different wavelengths.

To demonstrate the sensing performance and calibrate the smallest detectable displacement, thermal noise is measured in vacuum at room temperature using an electrical spectrum analyzer while the input laser wavelength is set at 1540 nm. The measurement setup is illustrated in Fig. 5a. The resolution bandwidth is set at 1 Hz and results are averaged over 20 acquisitions. A bias voltage of 1.5 V is applied on the actuator in order to separate the mechanical modes of the two suspended beams in frequency (see Supplementary Note 1). A thermal motion peak, related to the unbiased beam, is found at 1.624 MHz, as shown in Fig. 5b, which corresponds well to the value of 1.59 MHz predicted by a finite-element simulation. The quality factor of the peak is 1100.

The mean square oscillation of the cantilever due to thermal Brownian motion can be expressed as $\langle z_{th}^{2}\rangle = \frac{{k_bT}}{{(m_{eff}\omega ^2)}}$, where *k*_*b*_ is the Boltzmann constant, T is the temperature and *m*_*eff*_ is the motional mass of the fundamental mechanical mode of the cantilever, defined as $m_{eff} = \int dV\rho \vert {\boldsymbol{r}} ({\boldsymbol{x}})\vert^{2}$, where $\rho$ is the density of InP. The normalized mode shape **r**(**x**) is calculated from a finite-element model from which the motional mass is estimated to be 470 pg. By integrating the area below the photocurrent spectral density peak, a relation between photocurrent and displacement is found, providing a sensitivity of *∂I/∂z* = 388 A/m. Using this relation the photocurrent noise floor translates into a displacement imprecision of 45 fm/Hz^1/2^, limited by the pick-up electrical noise in the setup, as we noticed that the noise floor outside the resonance is independent of the laser and on whether the probe is in contact with the device. This value, obtained here in a monolithic sensor with integrated read-out, is on par with specifications of conventional AFM systems based on deflecting cantilevers and position-sensitive detectors, and is therefore adequate for nanomechanical sensing applications. Due to the large optical bandwidth of the nanomechanical directional coupler, similar sensitivities can be obtained throughout an ultrawide wavelength range, as shown in Fig. 5c where the Brownian motion is measured with laser wavelengths varying from 1520 nm to 1600 nm. The sensitivity *∂I/∂z* reaches maximum at wavelength of 1540 nm and is still about 85% of that at 1600 nm. This makes nano-optomechanical sensing possible with a simple fixed-wavelength laser in the telecom band.

### Dynamic response of the displacement sensor and Allan deviation

**Fig. 6:**
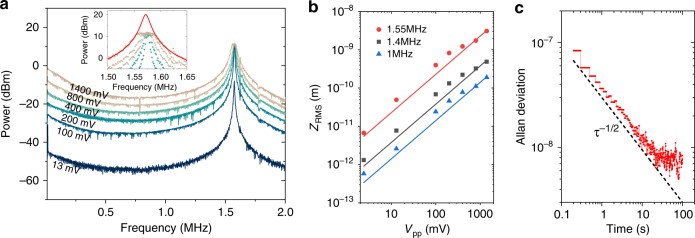
Response of the sensor driven by an oscillating voltage. **a** Driven-response spectra for various driving amplitude measured with a 50 Hz resolution bandwidth. A −1 V DC bias is applied for V_pp_ from 13 mV to 800 mV. For V_pp_ = 1.4 V a −1.4 V DC bias is used. Inset: Zoom-in of the spectra around at the resonance, with a Lorentzian fit of the 1.4 V modulation data. Laser wavelength is 1540 nm and on-chip power 0.3 mW. **b** Root mean square displacement of cantilever as a function of peak-to-peak voltage at 1.55 MHz, 1.4 MHz and 1 MHz. Dots are measurement data and solid lines are linear fits. **c** Allan deviation as a function of integration time. The τ^−1/2^ slope is indicated with dashed line, where τ is the integration time.

We further show the possibility to resonantly actuate and sense the membrane motion using the integrated actuator. The frequency response of the sensor is measured by driving the actuator using an AC signal with a sweeping frequency and recording the peak power in the power spectrum with an electric signal analyzer. A −1 V DC voltage is combined with the AC voltage through a bias-T to ensure that the actuator works in reverse bias. As can be seen from Fig. 6a, apart from a small initial decrease of the modulation amplitude in the 0–400 kHz frequency range, likely related to a surface-state induced lag effect^22^, the response is relatively flat until close to the mechanical resonance. As the AC driving voltage increases, the response becomes saturated at 11 dBm near the resonance, limited by the dynamic range of the transimpedance amplifier. A Lorentzian fit indicates the unsaturated peak power to be ∼20 dBm, corresponding to an oscillation peak-to-peak displacement of 32 nm. Together with the measured noise floor, this provides a potential displacement dynamic range >50 dB in a measurement bandwidth of 1 Hz. The driven displacement is calibrated using the sensitivity derived from the Brownian motion measurement and is plotted as a function of driving voltage in Fig. 6b, showing a linear relationship for frequencies both close to and away from the resonance.

The large dynamic range also enables a very precise measurement of the mechanical resonance frequency. The resonance frequency fluctuation is measured by driving the cantilever with an AC signal at the resonance frequency with peak-to-peak voltage of 400 mV. The Allan deviation is measured with an integration time from 0.2 s to 100 s using the electric signal analyzer and the result is shown in Fig. 6c. The slope follows an inverse square root dependence on the integration time, indicating that additive phase noise dominates the frequency stability^23^, until close to 100 s, where systematic drift starts to take place. For integration time of 1 s, a fluctuation of 0.056 Hz is obtained, corresponding to ${\mathrm{\Delta }}f/f_0 = 3.6 \, \times\,10^{ - 8}$. This high-frequency resolution may be instrumental for the demonstration of integrated sensors of particles and mass.

## Discussion

The main source of noise in our measurement comes from environmental electrical noise capacitively coupled to the metallic probe used to contact the diodes and the actuator. The stress induced by probe touching the contacts of actuator also causes the mechanical resonance of cantilever to shift by a few to tens of kHz. These issues could be improved by employing wire bonding in the future. The displacement noise floor measured in this work requires the laser intensity noise to be below −64 dB/Hz. A differential measurement of the two detection channels could further improve the tolerance to laser intensity noise. Displacement sensitivity and imprecision are expected to be improved significantly by enhancing the optical coupling efficiency through using an optical fiber instead of the objective to couple light into the waveguide. For the practical AFM applications some further steps are needed: a sharp dielectric tip can be grown on top of the cantilever using focused ion beam^24^, and the chip needs to be packaged into a holder with piezo motors to scan the sample. In this paper we have shown measurements in vacuum, and driving the actuator at atmospheric pressure will take a higher power due to the air damping. This will not affect contact-mode imaging, as $\partial I/\partial z$ is not expected to be affected by the pressure.

In summary, we have demonstrated an integrated optomechanical displacement sensor featuring 30 fm·Hz^−1/2^ imprecision along with >50 dB displacement dynamic range. The cavity-free design offers an ultrabroad optical bandwidth of >80 nm, eliminating the need for expensive tuneable lasers and making it immune to frequency noise. The on-chip waveguide photodiode replaces bulky free-space detectors for readout. The sensing concept and the used IMOS integration platform further lends itself to the integration of lasers^21,25^, paving the way to fully-integrated optomechanical sensors with electrical inputs/outputs for displacement and force sensing.

## Methods

### Experimental setup

Light from a tuneable laser (Santec TSL-510) was coupled into grating coupler (Grating1 in Fig. 2a) from the top through a × 50 objective (NA = 0.45). The coupling loss in this configuration is about 15 dB. The actuator and photodiodes were connected using two RF probes. In Figs. 2 and 3, the photocurrent was measured as a voltage drop on a 10 kΩ load resistor. For the measurements in Figs. 4 and 5, the photocurrent was amplified using a transimpedance amplifier (gain G = 5 × 10^5^ V/A). All measurements were performed at room temperature. Measurements in Figs. 3–5 were performed under vacuum condition (~10^−3^ mbar) to suppress viscous air damping.

### Fabrication process

Devices are fabricated on a InP/InGaAsP multilayer stack grown by metalorganic vapor-phase epitaxy (MOVPE) on an InP:Fe wafer. After removing the capping layers a 50-nm-thick SiN_x_ layer is deposited on the top of the wafer. Meanwhile, a silicon wafer is prepared and coated with 250 nm SiO_2_. Then the two wafers are bonded together with benzocyclobutene (BCB) at 280 ˚C in a wafer bonder. After bonding the InP substrate is etched away and a 100-nm-thick SiN_x_ layer is deposited as an etch mask. An electron beam lithography system is used to pattern the waveguides, actuators and detectors in resist which are then transferred into SiN_x_ using a CHF_3_ dry etch. Waveguides and other features are etched using a cyclic H_2_/CH_4_ and O_2_ etching process. An alloy of Ti-Pt-Au is used for both p- and n-contacts of the detector and the actuator. Devices are released by H_2_SO_4_:H_2_O_2_:H_2_O (1:1:10) wet etch at 20 °C to remove the sacrificial InGaAsP layer selectively from InP. Drying is performed with a CO_2_ critical point dryer to prevent stiction.

## Supplementary information

Supplementary Information

## Data Availability

All relevant data is available from the corresponding author upon reasonable request.
