# Author Correction: Integrated nano-optomechanical displacement sensor with ultrawide optical bandwidth

**DOI:** 10.1038/s41467-020-18579-2

**Published:** 2020-09-11

**Authors:** Tianran Liu, Francesco Pagliano, René van Veldhoven, Vadim Pogoretskiy, Yuqing Jiao, Andrea Fiore

**Affiliations:** 1 Institute for Photonic Integration, Eindhoven University of Technology, P.O. Box 513, 5600MB Eindhoven, The Netherlands; 2 nanoPHAB, Groene Loper 19, Postbus 513, 5612 AP Eindhoven, The Netherlands

**Keywords:** Integrated optics, Optical sensors

Correction to: *Nature Communications* 10.1038/s41467-020-16269-7, published online 15 May 2020.

The original version of this Article contained an error in the Discussion, which incorrectly read ‘In summary, we have demonstrated an integrated optomechanical displacement sensor featuring 30 fm·Hz^−1/2^ imprecision’. The correct version states ‘45’ in place of ‘30’. This has been corrected in both the PDF and HTML versions of the Article.

