# Supplementary material for: Integrated nano-optomechanical displacement sensor with ultrawide optical bandwidth
Source: Nat Commun. 2020 May 15;11:2407. doi: 10.1038/s41467-020-16269-7 (PMC7228956; doi:10.1038/s41467-020-16269-7)
Supplement: Supplementary file 1 — Supplementary Information [file 41467_2020_16269_MOESM1_ESM.pdf]

Supplementary Information for

Integrated Nano-Opto-Mechanical Displacement Sensor with Ultrawide Optical Bandwidth

Liu et al.

This PDF file includes:

Supplementary Note 1 and Supplementary Figure 1

Supplementary Note 1. Noise contribution from two independent oscillators

In the experiment of Fig. 5, both of the two suspended waveguides oscillate independently, results in two peaks (P1a and P1b) in the power spectral density (PSD), corresponding to their fundamental mechanical modes. Without external actuation, the two peaks almost overlap with each other due to their identical design. When one of the suspended waveguide is actuated electrostatically, the corresponded peak (P1a) shifts to lower frequency due to an electrostatic spring softening effect, while the unbiased one (P1b) remains at constant frequency, as shown in Fig. S1. In order to simplify the analysis, we chose the peak P1b at 1.5 V for the calibration of displacement imprecision in Fig. 5(b).

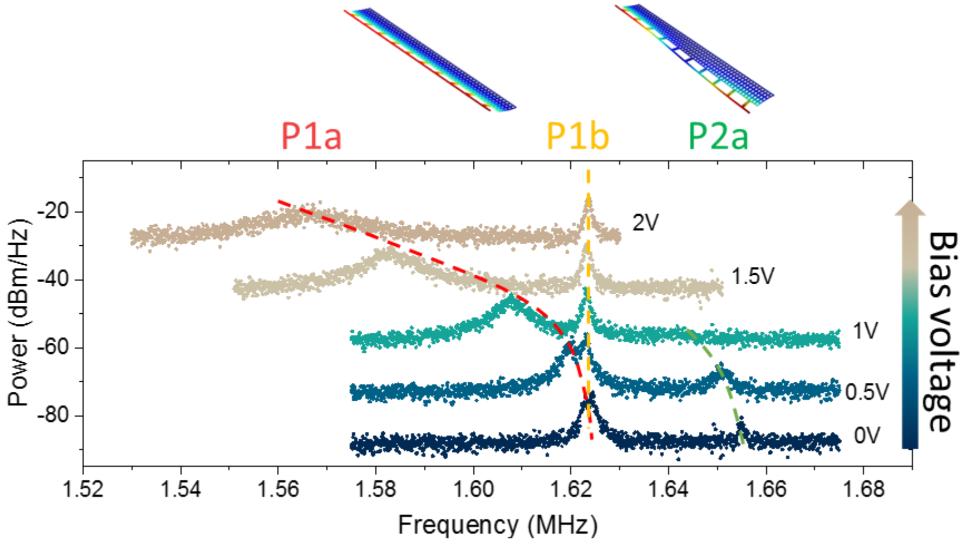

Supplementary Figure 1. Tuning PSD of thermal Brownian motion for different bias voltages on the actuator. The peak of the 1st mechanical mode of the biased cantilever (P1a) is marked by red dashed line, and that of the unbiased cantilever (P1b) is marked by yellow dashed line. The peak of the 2nd mechanical mode of the biased cantilever (P2a) is marked with green dashed line. Their simulated mode shape is shown above the graph.
